# *catena*-Poly[[tetra­aqua-μ-aqua-bis­(μ_4_-pyrimidine-2-carboxyl­ato)tetra­lithium] dichloride]

**DOI:** 10.1107/S1600536812041955

**Published:** 2012-10-20

**Authors:** Wojciech Starosta, Janusz Leciejewicz

**Affiliations:** a Institute of Nuclear Chemistry and Technology, ul.Dorodna 16, 03-195 Warszawa, Poland

## Abstract

The asymmetric unit of the title compound, [Li_4_(C_5_H_3_N_2_O_2_)_2_(H_2_O)_5_]Cl_2_, contains two Li^I^ cations, one with a distorted trigonal–bipyramidal and the other with a distorted tetra­hedral coordination geometry. Two symmetry-related asymmetric units constitute a building block of the structure, in which both ligand carboxyl­ate O atoms are bidentate and bridge the metal ions, forming a divalent cation. Charge balance is maintained by two chloride anions. The building blocks, bridged by Li^I^ cations, form cationic ribbons with chloride anions in the space between them. The ribbons propagate in [010] and are held together by a network of weak O—H⋯O hydrogen bonds which operate in the space between adjacent ribbons.

## Related literature

For the structure of a Li complex with pyrimidine-2-carboxyl­ate and nitrate ligands, see: Starosta & Leciejewicz (2011 ▶). The structure of a Li^I^ complex with pyrimidine-4-carboxyl­ate and water ligands was reported recently by Starosta & Leciejewicz (2012 ▶).
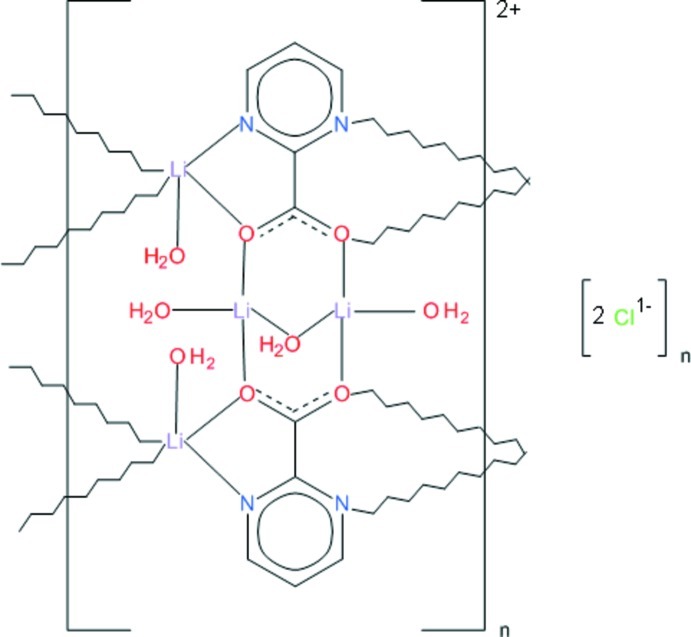


## Experimental

### 

#### Crystal data

[Li_4_(C_5_H_3_N_2_O_2_)_2_(H_2_O)_5_]Cl_2_
*M* *_r_* = 434.93
Monoclinic, 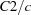

*a* = 22.084 (4) Å
*b* = 8.0773 (16) Å
*c* = 10.814 (2) Å
β = 94.08 (3)°
*V* = 1924.1 (7) Å^3^
*Z* = 4
Mo *K*α radiation
μ = 0.39 mm^−1^
*T* = 293 K
0.37 × 0.24 × 0.18 mm

#### Data collection

Kuma KM4 four-circle diffractometer
Absorption correction: analytical (*CrysAlis RED*; Oxford Diffraction, 2008 ▶) *T*_min_ = 0.917, *T*_max_ = 0.935
2964 measured reflections
2819 independent reflections
1781 reflections with *I* > 2σ(*I*)
*R*_int_ = 0.029
3 standard reflections every 200 reflections intensity decay: 2.3%

#### Refinement

*R*[*F* ^2^ > 2σ(*F* ^2^)] = 0.044
*wR*(*F* ^2^) = 0.147
*S* = 1.02
2819 reflections
152 parameters
4 restraints
H atoms treated by a mixture of independent and constrained refinement
Δρ_max_ = 0.51 e Å^−3^
Δρ_min_ = −0.36 e Å^−3^

### 

Data collection: *KM-4 Software* (Kuma, 1996 ▶); cell refinement: *KM-4 Software*; data reduction: *DATAPROC* (Kuma, 2001 ▶); program(s) used to solve structure: *SHELXS97* (Sheldrick, 2008 ▶); program(s) used to refine structure: *SHELXL97* (Sheldrick, 2008 ▶); molecular graphics: *SHELXTL* (Sheldrick, 2008 ▶); software used to prepare material for publication: *SHELXTL*.

## Supplementary Material

Crystal structure: contains datablock(s) I, global. DOI: 10.1107/S1600536812041955/qm2084sup1.cif

Structure factors: contains datablock(s) I. DOI: 10.1107/S1600536812041955/qm2084Isup2.hkl

Additional supplementary materials:  crystallographic information; 3D view; checkCIF report

## Figures and Tables

**Table 1:** Selected bond lengths (Å)

Li1—O1	2.069 (3)
Li1—N1	2.175 (4)
Li1—O3	1.987 (4)
Li1—O2^i^	2.029 (4)
Li1—N3^i^	2.221 (4)
Li2—O21	2.140 (6)
Li2—O22	1.915 (4)
Li2—O2^ii^	1.968 (4)
Li2—O1	1.993 (4)

Symmetry codes: (i) 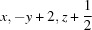; (ii) 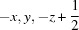.

**Table 2:** Hydrogen-bond geometry (Å, °)

*D*—H⋯*A*	*D*—H	H⋯*A*	*D*⋯*A*	*D*—H⋯*A*
O22—H222⋯O3	0.89 (2)	2.16 (3)	2.841 (3)	133 (3)
O22—H221⋯Cl1^iii^	0.87 (2)	2.36 (2)	3.212 (2)	167 (3)
O3—H31⋯Cl1	0.83 (4)	2.35 (4)	3.1381 (19)	158 (3)
O3—H32⋯Cl1^iv^	0.86 (4)	2.29 (4)	3.1429 (19)	168 (4)
O21—H211⋯Cl1^v^	0.84 (3)	2.45 (3)	3.288 (2)	175 (3)

Symmetry codes: (iii) 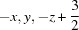; (iv) 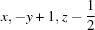; (v) 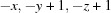.

**Fig. 1.**
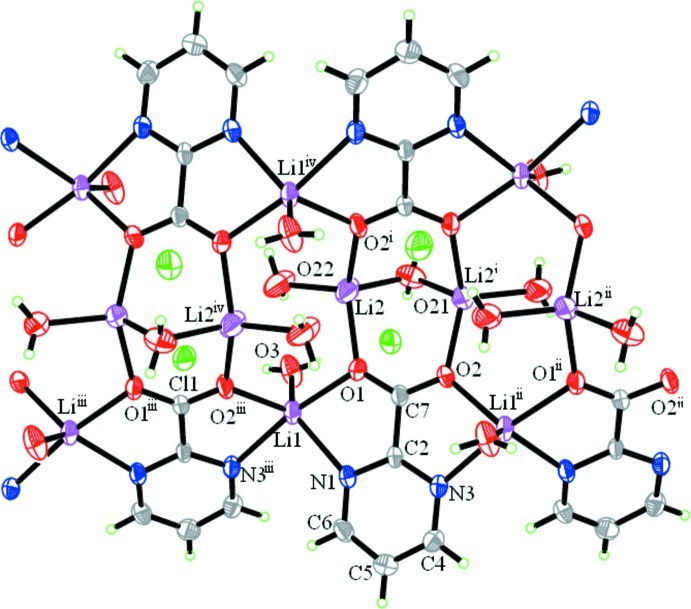
A fragment of a cationic ribbon of the title compound with atom labelling scheme and 50% probability displacement ellipsoids. Symmetry code: i -x, y, -z + 1/2; iix, -y + 2, z - 1/2; iiix, -y + 2, z + 1/2; iv -x, -y + 2, z + 1.

**Fig. 2.**
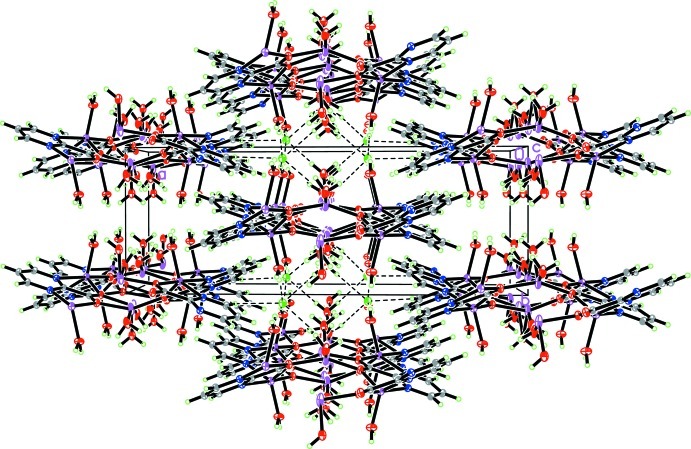
The alignment of cationic ribbons and chloride anions in the structure of the title compound viewed along the unit cell b direction.

## supplementary crystallographic information

### Comment

The asymmetric cell of the title compound contains two symmetry independent Li^I^ ions, one deprotonated pyramidine-2-carboxylatato ligand molecule, three symmetry independent water molecules which are coordinated to metal ions and one chloride anion. The latter maintains charge balance in the cell. Two symmetry related cells form a molecular moiety which can be visualized as a building unit of the structure (Fig. 1).The ligand bridges Li ions in a µ_4_ mode using both its carboxylate O atoms which act as bidentate. Ligand bonding groups N1,O1 and N3,O2 chelate Li1 and Li^ii^ ions; the O1 and O2 atoms are also bonded to Li2 and Li2^i^ ions, respectively. Since the Li1 and Li1^ii^ ions are also coordinated by bonding groups from adjacent symmetry related ligands, a –Li1^ii^—O2—O1—Li1—O2^iii^O1^iii^—Li1^iii^– bridging pathway is formed. Apart from two N,*O* bonding groups, Li1 coordination is completed by an aqua O3 atom. On the other hand, pairs of adjacent symmetry related Li2 and Li2^i^ ions are bridged by an aqua O21 atom while the other coordinated to them aqua O22 and O22^i^ atoms are not bridging. Symmetry code: ^i^ -*x*, *y*, -*z* + 1/2; ^ii^*x*, -*y* + 2, *z* - 1/2; ^iii^*x*, -*y* + 2, *z* + 1/2. Adjacent moieties linked along the Li1 bridging pathway form a cationic ribbon propagating in the unit cell *b* direction (Fig. 2). Chloride anions are located in the space between adjacent ribbons. Fig. 1 shows, that a ribbon can be visualized as built of centro-symmetric molecular clusters in which Li ions form a tetrameric entities additionally connected by Li2—O21—Li2^i^ bridges. The Li1 ion exhibits a distorted trigonal bipyramidal coordination environment in which O1, O3 and N3^iii^ atoms form an equatorial plane with a Li1 ion 0.0619 (2) Å out of it; N1 and O2^iii^ atoms are at apical positions. The coordination of the Li2 ion is strongly distorted tetrahedral. The Li—O and Li—N bond distances (Table 1) are close to those reported in the structures of a Li complex with the title and nitrate ligands (Starosta & Leciejewicz, 2011) and pyrimidine-4-carboxylate and water ligands (Starosta & Leciejewicz, 2012). The pyrimidine ring is planar with r.m.s. of 0.0071 (1) Å; the C7/O1/O2 makes with it a dihedral angle of 5.8 (1)°. Weak hydrogen bonds in which water O atoms are as donors and chloride anions act as acceptors operate between adjacent ribbons (Table 2).

### Experimental

1 mmol of methyl pyrimidine-2-carboxylate and *ca*2 mmol s of lithium hydroxide dissolved in 50 ml of hot, doubly distilled water were boiled under reflux with stirring for twenty hours. After evaporation to dryness at room temperature the polycrystalline material was dissolved in 50 ml of water. The solution was titrated with 1 N HCl until the pH reached 6.0 and then stirred for 3 h at *ca* 320 K. Left to crystallize at room temperature, colourless single-crystal blocks deposited after a week. They were washed with cold methanol and dried in the air.

### Refinement

Hydrogen atoms belonging to water molecules were located in a difference map and refined isotropically, while three H atoms attached to pyrimidine C atoms were located at calculated positions and treated as riding on the parent atoms with C—H=0.93 Å and *U*_iso_(H)=1.2*U*_eq_(C).

### Crystal data

**Table:**

[Li_4_(C_5_H_3_N_2_O_2_)_2_(H_2_O)_5_]Cl_2_	*F*(000) = 888
*M**_r_* = 434.93	*D*_x_ = 1.501 Mg m^−^^3^
Monoclinic, *C*2/*c*	Mo *K*α radiation, λ = 0.71073 Å
Hall symbol: -C 2yc	Cell parameters from 25 reflections
*a* = 22.084 (4) Å	θ = 6–15°
*b* = 8.0773 (16) Å	µ = 0.39 mm^−^^1^
*c* = 10.814 (2) Å	*T* = 293 K
β = 94.08 (3)°	Block, colourless
*V* = 1924.1 (7) Å^3^	0.37 × 0.24 × 0.18 mm
*Z* = 4	

### Data collection

**Table:**

Kuma KM4 four-circle diffractometer	1781 reflections with *I* > 2σ(*I*)
Radiation source: fine-focus sealed tube	*R*_int_ = 0.029
Graphite monochromator	θ_max_ = 30.1°, θ_min_ = 1.9°
profile data from ω/2θ scans	*h* = −31→31
Absorption correction: analytical (*CrysAlis RED*; Oxford Diffraction, 2008)	*k* = −11→0
*T*_min_ = 0.917, *T*_max_ = 0.935	*l* = 0→15
2964 measured reflections	3 standard reflections every 200 reflections
2819 independent reflections	intensity decay: 2.3%

### Refinement

**Table:**

Refinement on *F*^2^	Primary atom site location: structure-invariant direct methods
Least-squares matrix: full	Secondary atom site location: difference Fourier map
*R*[*F*^2^ > 2σ(*F*^2^)] = 0.044	Hydrogen site location: inferred from neighbouring sites
*wR*(*F*^2^) = 0.147	H atoms treated by a mixture of independent and constrained refinement
*S* = 1.02	*w* = 1/[σ^2^(*F*_o_^2^) + (0.0879*P*)^2^ + 1.2478*P*] where *P* = (*F*_o_^2^ + 2*F*_c_^2^)/3
2819 reflections	(Δ/σ)_max_ = 0.004
152 parameters	Δρ_max_ = 0.51 e Å^−^^3^
4 restraints	Δρ_min_ = −0.36 e Å^−^^3^

### Special details

**Table:**

Geometry. All e.s.d.'s (except the e.s.d. in the dihedral angle between two l.s. planes) are estimated using the full covariance matrix. The cell e.s.d.'s are taken into account individually in the estimation of e.s.d.'s in distances, angles and torsion angles; correlations between e.s.d.'s in cell parameters are only used when they are defined by crystal symmetry. An approximate (isotropic) treatment of cell e.s.d.'s is used for estimating e.s.d.'s involving l.s. planes.
Refinement. Refinement of *F*^2^ against ALL reflections. The weighted *R*-factor *wR* and goodness of fit *S* are based on *F*^2^, conventional *R*-factors *R* are based on *F*, with *F* set to zero for negative *F*^2^. The threshold expression of *F*^2^ > σ(*F*^2^) is used only for calculating *R*-factors(gt) *etc*. and is not relevant to the choice of reflections for refinement. *R*-factors based on *F*^2^ are statistically about twice as large as those based on *F*, and *R*- factors based on ALL data will be even larger.

### Fractional atomic coordinates and isotropic or equivalent isotropic displacement parameters (Å^2^)

**Table:**

	*x*	*y*	*z*	*U*_iso_*/*U*_eq_	
O1	0.08301 (6)	0.92604 (19)	0.37614 (11)	0.0321 (3)	
O2	0.08768 (6)	0.96441 (19)	0.17202 (11)	0.0319 (3)	
N3	0.20103 (7)	1.0854 (2)	0.20733 (13)	0.0277 (3)	
N1	0.19897 (7)	1.0170 (2)	0.42115 (13)	0.0277 (3)	
C6	0.25599 (9)	1.0720 (3)	0.43984 (17)	0.0326 (4)	
H6	0.2752	1.0657	0.5191	0.039*	
C2	0.17410 (7)	1.0274 (2)	0.30494 (14)	0.0232 (3)	
C4	0.25768 (9)	1.1411 (3)	0.22901 (18)	0.0332 (4)	
H4	0.2778	1.1836	0.1634	0.040*	
C5	0.28729 (9)	1.1378 (3)	0.3457 (2)	0.0353 (4)	
H5	0.3266	1.1781	0.3601	0.042*	
Li1	0.13426 (15)	0.9162 (5)	0.5437 (3)	0.0323 (7)	
O3	0.11355 (9)	0.6795 (2)	0.56747 (16)	0.0469 (4)	
C7	0.10916 (8)	0.9673 (2)	0.28238 (14)	0.0239 (3)	
O21	0.0000	0.6868 (4)	0.2500	0.0527 (6)	
Li2	−0.00573 (17)	0.8841 (8)	0.3807 (4)	0.0590 (13)	
Cl1	0.10829 (3)	0.57231 (8)	0.84542 (5)	0.04391 (17)	
H211	−0.0286 (13)	0.622 (4)	0.230 (3)	0.064 (10)*	
H32	0.1130 (18)	0.599 (5)	0.515 (4)	0.086 (12)*	
H31	0.1170 (14)	0.627 (4)	0.634 (3)	0.065 (9)*	
O22	−0.00669 (8)	0.8066 (3)	0.54795 (18)	0.0618 (6)	
H221	−0.0386 (10)	0.754 (4)	0.570 (3)	0.075 (10)*	
H222	0.0166 (14)	0.717 (4)	0.555 (3)	0.120 (17)*	

### Atomic displacement parameters (Å^2^)

**Table:**

	*U*^11^	*U*^22^	*U*^33^	*U*^12^	*U*^13^	*U*^23^
O1	0.0302 (6)	0.0478 (8)	0.0185 (6)	−0.0032 (6)	0.0031 (5)	0.0039 (5)
O2	0.0313 (6)	0.0472 (8)	0.0164 (5)	−0.0051 (6)	−0.0040 (4)	0.0036 (5)
N3	0.0316 (7)	0.0335 (8)	0.0181 (6)	−0.0022 (6)	0.0028 (5)	0.0009 (6)
N1	0.0312 (7)	0.0352 (8)	0.0165 (6)	−0.0006 (6)	−0.0012 (5)	0.0012 (6)
C6	0.0330 (8)	0.0390 (10)	0.0245 (8)	−0.0006 (8)	−0.0062 (6)	−0.0016 (7)
C2	0.0291 (8)	0.0258 (8)	0.0148 (6)	0.0024 (6)	0.0012 (5)	0.0000 (6)
C4	0.0354 (9)	0.0361 (10)	0.0289 (9)	−0.0051 (8)	0.0075 (7)	0.0008 (7)
C5	0.0289 (9)	0.0392 (10)	0.0373 (10)	−0.0036 (8)	−0.0006 (7)	−0.0014 (8)
Li1	0.0376 (16)	0.0420 (18)	0.0173 (13)	0.0004 (14)	0.0020 (11)	−0.0006 (12)
O3	0.0754 (12)	0.0358 (8)	0.0277 (7)	0.0017 (8)	−0.0090 (7)	0.0005 (7)
C7	0.0258 (7)	0.0285 (8)	0.0171 (7)	0.0017 (6)	0.0006 (5)	0.0009 (6)
O21	0.0337 (11)	0.0675 (17)	0.0551 (15)	0.000	−0.0086 (10)	0.000
Li2	0.0301 (17)	0.116 (4)	0.0305 (18)	0.009 (2)	0.0030 (14)	0.018 (2)
Cl1	0.0420 (3)	0.0553 (3)	0.0345 (3)	−0.0001 (2)	0.00346 (19)	0.0087 (2)
O22	0.0349 (8)	0.0967 (16)	0.0550 (11)	0.0101 (10)	0.0123 (7)	0.0338 (11)

### Geometric parameters (Å, º)

**Table:**

O1—C7	1.247 (2)	Li1—N3^iii^	2.221 (4)
Li1—O1	2.069 (3)	Li1—Li2^iv^	3.414 (6)
O2—C7	1.2528 (19)	O3—H32	0.86 (4)
O2—Li2^i^	1.968 (4)	O3—H31	0.83 (4)
O2—Li1^ii^	2.029 (4)	Li2—O21	2.140 (6)
N3—C2	1.333 (2)	O21—Li2^i^	2.140 (6)
N3—C4	1.335 (2)	O21—H211	0.84 (3)
N3—Li1^ii^	2.221 (4)	Li2—O22	1.915 (4)
N1—C6	1.337 (2)	Li2—O2^i^	1.968 (4)
N1—C2	1.338 (2)	Li2—O1	1.993 (4)
Li1—N1	2.175 (4)	Li2—O22^iv^	2.623 (7)
C6—C5	1.377 (3)	Li2—Li2^i^	2.856 (8)
C6—H6	0.9300	Li2—Li2^iv^	3.183 (11)
C2—C7	1.517 (2)	Li2—Li1^iv^	3.414 (6)
C4—C5	1.379 (3)	Li2—H222	2.343 (18)
C4—H4	0.9300	O22—Li2^iv^	2.623 (7)
C5—H5	0.9300	O22—H221	0.868 (18)
Li1—O3	1.987 (4)	O22—H222	0.891 (18)
Li1—O2^iii^	2.029 (4)		
			
C7—O1—Li2	125.36 (16)	Li2—O21—Li2^i^	83.7 (3)
C7—O1—Li1	117.79 (15)	Li2—O21—H211	124 (2)
Li2—O1—Li1	116.70 (16)	Li2^i^—O21—H211	112 (2)
C7—O2—Li2^i^	124.07 (17)	O22—Li2—O2^i^	108.02 (19)
C7—O2—Li1^ii^	117.82 (15)	O22—Li2—O1	98.98 (19)
Li2^i^—O2—Li1^ii^	117.32 (18)	O2^i^—Li2—O1	145.3 (3)
C2—N3—C4	116.42 (15)	O22—Li2—O21	112.7 (3)
C2—N3—Li1^ii^	108.69 (14)	O2^i^—Li2—O21	98.6 (2)
C4—N3—Li1^ii^	134.65 (15)	O1—Li2—O21	90.32 (18)
C6—N1—C2	116.14 (15)	O22—Li2—O22^iv^	92.4 (2)
C6—N1—Li1	133.22 (14)	O2^i^—Li2—O22^iv^	81.1 (2)
C2—N1—Li1	110.62 (14)	O1—Li2—O22^iv^	76.29 (19)
N1—C6—C5	122.22 (17)	O21—Li2—O22^iv^	153.3 (2)
N1—C6—H6	118.9	O22—Li2—Li2^i^	160.4 (2)
C5—C6—H6	118.9	O2^i^—Li2—Li2^i^	81.81 (19)
N3—C2—N1	126.11 (16)	O1—Li2—Li2^i^	79.56 (18)
N3—C2—C7	117.06 (14)	O21—Li2—Li2^i^	48.13 (14)
N1—C2—C7	116.82 (15)	O22^iv^—Li2—Li2^i^	106.04 (10)
N3—C4—C5	122.10 (17)	O22—Li2—Li2^iv^	55.42 (15)
N3—C4—H4	119.0	O2^i^—Li2—Li2^iv^	93.4 (2)
C5—C4—H4	119.0	O1—Li2—Li2^iv^	84.2 (2)
C6—C5—C4	116.98 (18)	O21—Li2—Li2^iv^	165.6 (3)
C6—C5—H5	121.5	O22^iv^—Li2—Li2^iv^	36.95 (15)
C4—C5—H5	121.5	Li2^i^—Li2—Li2^iv^	142.6 (2)
O3—Li1—O2^iii^	103.68 (16)	O22—Li2—Li1^iv^	82.13 (16)
O3—Li1—O1	91.92 (15)	O2^i^—Li2—Li1^iv^	31.87 (10)
O2^iii^—Li1—O1	107.96 (16)	O1—Li2—Li1^iv^	139.6 (3)
O3—Li1—N1	127.32 (18)	O21—Li2—Li1^iv^	126.81 (17)
O2^iii^—Li1—N1	128.74 (19)	O22^iv^—Li2—Li1^iv^	63.32 (13)
O1—Li1—N1	78.11 (12)	Li2^i^—Li2—Li1^iv^	111.75 (17)
O3—Li1—N3^iii^	92.14 (15)	Li2^iv^—Li2—Li1^iv^	63.10 (15)
O2^iii^—Li1—N3^iii^	78.36 (12)	O22—Li2—H222	21.2 (6)
O1—Li1—N3^iii^	171.42 (18)	O2^i^—Li2—H222	124.0 (7)
N1—Li1—N3^iii^	93.42 (14)	O1—Li2—H222	88.1 (8)
O3—Li1—Li2^iv^	102.75 (17)	O21—Li2—H222	94.7 (9)
O2^iii^—Li1—Li2^iv^	30.81 (10)	O22^iv^—Li2—H222	107.6 (10)
O1—Li1—Li2^iv^	77.24 (13)	Li2^i^—Li2—H222	140.1 (7)
N1—Li1—Li2^iv^	124.23 (17)	Li2^iv^—Li2—H222	71.8 (9)
N3^iii^—Li1—Li2^iv^	109.16 (14)	Li1^iv^—Li2—H222	102.2 (6)
Li1—O3—H32	129 (3)	Li2—O22—Li2^iv^	87.6 (2)
Li1—O3—H31	126 (2)	Li2—O22—H221	119 (2)
H32—O3—H31	101 (3)	Li2^iv^—O22—H221	117 (2)
O1—C7—O2	127.12 (16)	Li2—O22—H222	107.6 (17)
O1—C7—C2	116.22 (14)	Li2^iv^—O22—H222	135 (3)
O2—C7—C2	116.65 (15)	H221—O22—H222	93 (2)

Symmetry codes: (i) −*x*, *y*, −*z*+1/2; (ii) *x*, −*y*+2, *z*−1/2; (iii) *x*, −*y*+2, *z*+1/2; (iv) −*x*, −*y*+2, −*z*+1.

### Hydrogen-bond geometry (Å, º)

**Table:**

*D*—H···*A*	*D*—H	H···*A*	*D*···*A*	*D*—H···*A*
O22—H222···O3	0.89 (2)	2.16 (3)	2.841 (3)	133 (3)
O22—H221···Cl1^v^	0.87 (2)	2.36 (2)	3.212 (2)	167 (3)
O3—H31···Cl1	0.83 (4)	2.35 (4)	3.1381 (19)	158 (3)
O3—H32···Cl1^vi^	0.86 (4)	2.29 (4)	3.1429 (19)	168 (4)
O21—H211···Cl1^vii^	0.84 (3)	2.45 (3)	3.288 (2)	175 (3)

Symmetry codes: (v) −*x*, *y*, −*z*+3/2; (vi) *x*, −*y*+1, *z*−1/2; (vii) −*x*, −*y*+1, −*z*+1.
